# Mental health hygiene during a health crisis: Exploring factors associated with media-induced secondary trauma in relation to the COVID-19 pandemic

**DOI:** 10.1177/20551029231199578

**Published:** 2023-09-22

**Authors:** Nishtha Lamba, Olga Khokhlova, Aditi Bhatia, Cillian McHugh

**Affiliations:** 1 Department of Psychology, 156575 Middlesex University Dubai, Dubai, UAE; 2 Department of Psychology, 8808 University of Limerick, Limerick, Ireland

**Keywords:** pandemic, paranoia, media trauma, conspiracy theories, public health measures, compliance, news media

## Abstract

**Aims:**

Given the risk of developing vicarious trauma through news media has increased during the pandemic, we explored risk factors associated with media induced secondary trauma, and its behavioral and psychological implications.

**Methods:**

An international study (*N* = 1066), with a diverse sample, was administered in July 2020. We used standardized and validated questionnaires to measure news consumption, media-related trauma, compliance, and paranoia.

**Results:**

Greater frequency of news consumption, accessing news via social media and WHO, and believing in conspiracy theories increased likelihood of developing media-induced secondary trauma. News related trauma was associated with greater compliance with safety measures and increased paranoid ideation. Media-trauma however exhibited a greater association with paranoia than compliance.

**Conclusion:**

Findings highlight the need to facilitate a collaborative intervention, with public, media houses, health safety officials, and social scientists to have a deeper understanding of potential psychological costs of news consumption patterns.

## Introduction

During the pandemic, news media, in all its forms, has been providing constant COVID-19 related updates on number of cases and deaths, theories about the origin of the virus, safety guidelines, and new medical research findings. News media consumption patterns, both in terms of frequency of use and sources, could have a negative psychological and behavioral effect on consumers (Moghanibashi-Mansourieh, 2020, Olagoke et al., 2020, Purohit et al., 2018).

Unhealthy patterns of media consumption have been associated with secondary trauma (Comstock and Platania, 2017). Based on the theory of vicarious traumatization (Molnar et al., 2017, Sui and Padmanabhanunn, 2016), the present study explores the impact of secondary trauma experience through news media during the pandemic. Previous research has demonstrated vicarious trauma in people with high levels of exposure to primary traumatic experiences of other individuals. For instance, in the aftermath of 9/11 in New York or 2013 Boston Marathon Bombings, researchers found that both the quantity and intensity of the visually graphic nature of the trauma reported in media, was associated with poor mental health, or even PTSD (Holman et al., 2020, Ahern et al., 2002). Applied to the context of the pandemic, repeated exposure to media reports of community crisis, or highlighting the threat of death and bereavement (including footage of crowded hospital conditions, patients on ventilators, but also mortality statistics, etc.), could lead to increase in stress and anxiety (Mensi et al., 2021). Intrusive imagery contributes to health anxiety and in some cases even anxiety disorders (Muse et al., 2010). Given the regular claims about the health system's collapse (Collado-Boira et al., 2020, da Silva and Pena, 2021, Rocha et al., 2021), it seems reasonable to assume that these images were rather anxiety provoking. This could potentially affect even those who are otherwise at a low risk of contracting the virus (Garfin et al., 2020). Indeed, recent research has shown that frequent exposure to mortality statistics and fearful messaging related to illness and death during the pandemic increased ‘coronaphobia’, leading to higher rates of generalized anxiety, death anxiety, depression, and insomnia, and even changes in adherence to safety behaviors (Lee et al., 2020).

Secondary trauma within the context of the large-scale news coverage of traumatic events (such as we have seen with the coronavirus pandemic), has not been widely studied (see Molnar et al., 2017). Vicarious or secondary trauma may lead to symptoms of hypervigilance, including frequent monitoring of news, hyperawareness of possible exposure to the virus, increased bodily anxiety, nightmares, or disturbed sleep (Chan et al., 2020, Gupta, 2020). Symptoms of such secondary trauma bears resemblance to Post Traumatic Stress Disorder. Coronavirus related vivid imagery of hospital beds (signaling death and bereavement), viruses, mortality statistics etc. (Usher et al., 2020), could also trigger such symptoms.

The way we access news media impacts how we engage with it. In recent years, social media (e.g., Twitter, Facebook, etc.) has provided an important *platform for accessing news media*. A study recently conducted in Wuhan, China, found that frequent social media use during the pandemic was associated with higher anxiety in the public (Gao et al., 2020). However, questions have been raised about the accuracy and impact of news sources accessed via social media leading to increased pressure on these platforms to fact-check the information that is being circulated (Ahmed et al., 2020, Pennycook et al., 2020).

Inconsistent or contradictory information in news during the pandemic may lead to public suspicion regarding the *credibility of the pandemic* (Coscieme et al., 2020), negatively impacting people’s faith in media and response to, and even compliance with governmental measures (Chao et al., 2020). Research during H1*N*1 pandemic in 2009, demonstrated that lower *trust in media*, along with perceived media exaggeration, had a negative impact on public’s mental health and compliance attitude (Prati et al., 2011). In addition, several *conspiracy theories* often based on biased or extremist media reports have been widely circulated (Allington et al., 2020, Calvillo et al., 2020). Previous research has shown that people can vary in their susceptibility to believe conspiracy theories (Brotherton et al., 2013), and exposure to conspiracy theories through media and social media influences people I different ways. Thus, in addition to investigating frequency and type of news media consumption, we also investigate the influence of general conspiracy beliefs.

While it is known that belief in conspiracies influences compliance with health regulations and increases mistrust and paranoia in the public (Freeman et al., 2020), the specific behavioral and psychological implications regarding media related secondary trauma are less understood. Cognitive factors such as critical thinking have been shown to be related to how people engage with, and how people might be affected by their engagement with news media. For instance, previous research has shown that lower levels of critical thinking are associated with higher media-related trauma (Pinchevski, 2016, Trnka and Lorencova, 2020). In the current research we extend this existing work offering novel insights from diverse international samples. In line with previous findings (Pinchevski, 2016, Trnka and Lorencova, 2020), we hypothesize that in our study media-related secondary trauma will be associated with lower levels of critical thinking.

An additional potential consequence of the pandemic, and the consumption of news related to the pandemic is an increase in paranoia within the public. Previous research has reported an increase in *paranoia* during the coronavirus pandemic (Larsen et al., 2020), however the impact of news consumption patterns on paranoia, has not been directly studied. We address this by hypothesizing that news related trauma during the pandemic will be associated with increased paranoia.

It is also unclear if these fear-based news messaging contributes towards *compliance* to safety measures. Recent research administered in Italy, suggests that greater media exposure during the pandemic increased both protection-based behaviors and state anxiety (Rubaltelli et al., 2020). Their study, focused on media exposure and consumption, here, we build on this by investigating media induced secondary trauma and its possible influence on both compliance and paranoia. So, overall while a growing body of research has examined the overall physical and psychological implications of the pandemic (Arora and Grey, 2020, Ingram et al., 2020, Ingram et al., 2021, Ingram et al., 2022, Odriozola-Gonzalez et al., 2020, Zysberg and Zisberg, 2020), there is lack of empirical research exploring the psychological and behavioral implications of news media during the pandemic.

The aim of the study is to explore risk factors associated with news media related secondary trauma, and behavioral and psychological implications of such potential secondary trauma during the pandemic. We study various influencing factors in social ecological systems such as demographics, attitudes, beliefs, and institutional trust to understand vicarious trauma through news media (Napoli et al., 2021). Given the international nature of the pandemic, we explored media trauma at a global level. We first explore the frequency of news media consumption, identify preferred media sources for COVID-19 related information and assess whether these are related to greater media-induced secondary trauma. Second, after accounting for demographic characteristics (age, gender, and educational qualifications), attitudes towards media (measured by faith in media), and belief in the credibility of the pandemic (measured by perceived seriousness of the pandemic), the current research examines a) the impact of cognitive beliefs (belief in conspiracy theories) and cognitive resources (critical thinking) on perceived media-related trauma b) the impact of perceived media-related trauma on compliance to safety regulations during the pandemic, and c) the impact of perceived media related secondary trauma on levels of paranoia in the public.

## Materials and methods

### Participants and procedure

We posted a survey link on platforms – Reddit, WhatsApp, Facebook, Instagram, and other online blogs. Priori analysis using G*Power suggested that to achieve a desired power of .80 and p value less than 0.05 for two tailed tests, we required a minimum of 72 participants to detect a large effect size (0.35), a sample of *N* = 103 to detect a medium effect size (0.15), and a minimum of 725 participants to detect a small effect size of 0.02 (Faul et al., 2007, 2009).

Prospective sample sizes were modelled out based on reasonable large, medium and small effect sizes. There are two main approaches to determining if the effect size is meaningful or not. The first approach suggests that researchers need to compare the effect found in a study with the effects found in previous studies in the respective area of research. Studies conducted on trauma and reaction to media exposure to distress usually report effect sizes of Hedges’ g around 1.19 - 1.61 (Hopwood and Schutte, 2017, Rubin et al., 2017), which are large effect sizes. However, these studies have quite small sample sizes (Hopwood and Schutte, 2017), and as discussed in the literature, small sample size studies produce larger effect sizes than large studies (Slavin and Smith, 2009). Given that the current study was exploratory in nature and requires a large sample size, it was decided to apply the second approach, which is a use of global conventional benchmarks for small, medium, and large effects. Thus, we expect the effect sizes to be rather small (Sullivan and Feinn, 2012). Our a priori power analyses were based on the overall effect size for the full regression model, in order to ensure sufficient statistical power to make meaningful inferences regarding the individual predictors in the models, we set our target minimum sample size as sufficient to detect a small effect.

So the total sample size was finalized based on feasibility of data collection and research studies of similar design and scale. Given the global nature of this pandemic we chose to collect international level data. The distribution of data provides information from eight countries, with a wide representation from the Global South.

The study survey was initially completed by 1083 adult participants residing in 50 countries in Asia, Europe, North America, and Australia. 17 participants were removed at the time of screening as they did not consent to the study, were younger than 18, or failed the test for duplicate cases. Final sample consisted of 1066 participants (651 female, 409 male, three prefer not to answer, *M*_*age*_: 33.51, *SD*_*age*_ = 12.43, range_age_ = 18 – 83). Majority of the participants were young adults, between the age of 18-27 (*n* = 404). Rest of the participants had the following age distribution: 28-37 (*n* = 299), 38-47 (*n* = 166), 48-57 (*n* = 104), 58-67 (*n* = 39), 68-77 (*n* = 10), 78-83 (*n* = 4).

Top eight countries, (India, *n* = 321; Russia, *n* = 271; USA, *n* = 108; UK, *n* = 79; Canada, *n* = 48; Germany, *n* = 41; Australia, *n* = 31; Pakistan, *n* = 30), with at least 30 responses each, were used to explore country level comparisons (see supplemental material table I). Educational status was as follows: Doctoral-Level Qualification (5.5%), Postgraduate qualification (45.2%), Undergraduate qualification (32.6%) and High School Diploma or lower (16.2%). We used a combination of convenience and snowball sampling, and recruited a diverse representation of participants, in terms of the size of the outbreak. Participants provided informed consent and the study was approved by the ethics committee.

## Materials

### Standardized questionnaires

#### Media-induced secondary trauma scale

(Comstock and Platania, 2017): This scale was adapted to measure symptoms of perceived secondary traumatic stress experienced after repeated exposure of real-life traumas through news media during COVID-19. Participants were asked to reflect on the pandemic when answering the questions. To our knowledge, there are no other standardized questionnaires to assess media-generated secondary trauma. In line with the questionnaire, survey-based questions regarding media usage patterns were added in the beginning. Participants were asked about their frequency and sources of news media consumption to access COVID-19 related information: “*Nowadays, how many hours per day are you exposed to news or learning about world events, especially related to COVID-19*?” “*From which source do you mostly get your information about the pandemic?”*. Questionnaire has 16 items. Example is - *“In the past SEVEN (7) days, after being exposed to others’ real-life trauma(s) on television and/or through social media, I felt emotionally numb”*, 1 = Never, and 5 = Very Often; Cronbach alpha based on the original scale administered in the study was α= 0.93.

#### Critical thinking disposition scale

(Sosu, 2013): This scale assesses aspects like critical openness and reflective skepticism and has 11 items. A sample item is *“I usually check the credibility of the source of information before making judgements”*, 1 = strongly disagree, 5 = strongly agree. Cronbach alpha was α= 0.90. Original unedited scale was administered and Cronbach alpha is based on the current dataset.

#### Generic conspiracy beliefs scale

(Brotherton et al., 2013): The scale measured how likely participants are to hold general conspiracy beliefs. It consists of 15 items related to government malfeasance, extra-terrestrial cover-up, malevolent global conspiracies, personal wellbeing, and control of information. Sample item is *“Groups of scientists manipulate, fabricate, or suppress evidence in order to deceive the public”,* 1 = definitely not true, 5 = definitely true. Cronbach alpha was α = 0.94. Original unedited scale was administered and Cronbach alpha is based on the current dataset.

#### Paranoia scale

(Fenigstein and Vanable, 1992): The scale administered paranoid ideation. It has 20 items, for example, *“It is safer to trust no one”*, 1 = not at all applicable to me, 5 = extremely applicable to me. Cronbach alpha was α = 0.92. Original unedited scale was administered, and Cronbach alpha is based on the current dataset.

### Survey questions

A few one-item questions were added to the survey a) due to lack of suitable standardized questionnaires and b) to facilitate an appropriate duration of the survey.

#### Perceived seriousness of the pandemic

It was assessed by asking participants *“How serious do you think the situation with COVID-19 actually is”,* 1 = not serious at all, 5 = extremely serious.

#### Faith in media’s portrayal of the pandemic

We asked participants *“How accurately do you think the situation was portrayed by media*?”, 1 = not at all accurate, 5 = extremely accurate.

#### Perception of compliance with COVID-19 guidelines

It was measured by asking participants “*Generally, do you believe you are following safety guidelines more than others in your community?”*, 1 = much less than others, 5 = much more than others.

### Analytical approach

Data was analyzed using SPSS v26.0. Descriptive statistics were used to report participants’ responses to frequency of news media consumption during the pandemic and sources of media chosen for COVID-19 related information (see Table 1). Missing data was not provided any pseudo-numeric codes. Pearson bivariate correlation was then used to assess whether frequency and source of media consumption significantly relate to media-induced secondary trauma. After accounting for demographic characteristics and attitudes towards the pandemic and media, three hierarchical regressions were conducted a) to identify factors which may increase risk of media-induced trauma, and b) to assess the behavioral (compliance related behavior) and c) psychological (public’s level of paranoid ideation) implications of media-induced trauma. Variables were entered as per the research objectives and key variables of interest were added in the last step of the models. We accounted for demographic variables (primarily age, gender, and education) first. Relevant attitude towards the pandemic and media (seriousness towards the pandemic and fath in media) which may explain the DVs, were accounted for next in all three models. Key variables, identified based on previous literature and psychological theories (were selectively added in the last step of the model to observe whether it adds substantial value in explaining the DV. The larger focus was to observe change in predictability based on predictor variables added at the end of the analysis (Petrocelli, 2003). The regressions do not imply causality.

**Table 1.** Media consumption patterns.

Variables		Frequency %
Frequency of media consumption	Several times per hour	4.2
	Several times per day	33.7
	Once a day	27.2
	Once every couple of days	20.3
	Approx. Once a month	12.1
	Not at all	3.2
Sources of media consumption^a^	Social media	62.2
	Online news	61.3
	Television	41.5
	World health organization	16.3
	Government websites	18.9
	Radio	7.1

^a^Participants chose multiple options.

## Results

### Descriptive statistics and inter-correlations

Greater frequency of consumption of pandemic related news was positively correlated with media-induced secondary trauma *r*(955) = 0.14, *p* < .001. Social media, *r*(955) = 0.16, *p* < .001, WHO *r*(955) = 0.07, *p* = .02, and Radio *r*(955) = -0.07, *p* = .02 were significantly related to media-induced secondary trauma (see supplementary table for country level data).

While access to WHO did not significantly vary based on educational status (χ2 = 4.08, *p* = .53), younger people were more likely to access news via WHO significantly (r = -0.09, *p* = .003).

The correlation matrix of the variables is presented in Table 2. Findings will be discussed with caution as given a large sample size, there are a few significant yet weak correlations in the data.

**Table 2.** Correlations and mean scores of main study variables.

	Variable	1	2	3	4	5	6	7	8
1	Media-induced trauma								
2	Critical thinking	.10**							
3	General conspiracy beliefs	.24***	.13***						
4	Paranoia	.37***	.11**	.43***					
5	Media consumption	.14***	.04	-.10**	.09**				
6	Faith in media	-.01	-.01	-.20***	-.03	.30***			
7	Compliance with guidelines	.21***	.07*	-.01	.07*	.30***	.26***		
8	Perceived seriousness of the situation	.20***	.04	-.13***	-.01	.29***	.39***	.43**	
	Mean (SD)	35.7 (13.6)	43.25 (7.45)	40.06 (13.55)	44.84 (16.12)	6.71 (1.94)	2.96 (1.12)	3.71 (1.02)	4.12 (.96)

*p* < .001***, *p* < .01**, *p* < .05*.

### Regressions

First hierarchical regression was administered to examine whether demographic variables (age, gender, and educational level; entered at step 1), perceived seriousness of the pandemic and faith in media (entered at step 2), and cognitive processes variables - levels of critical thinking and general conspiracy beliefs (entered at the last step) significantly predicted COVID-19 related media-induced secondary trauma. Collinearity statistics were checked using Tolerance (range = 0.81 – 0.97), and VIF (range = 1.02 – 1.2) values. The models were significant, explaining 19% variance. Demographic variables explained maximum variance (Adjusted R^2^ = 0.10) in media related trauma *F*(3, 819) = 32.5, *p* < .001. The second model explained an additional 4% variance *F*(5, 817) = 28.51, *p* < .001. Critical thinking and general belief in conspiracies explained an additional 5% (Adjusted *R*^2^ = 0.05) variance in media related trauma *F*(7, 815) = 28.87, *p* < .001. As shown in Table 3, participants who were younger, (*p* < .001), were females (*p* < .001), perceived pandemic to be more serious (*p* < .001) and scored higher on conspiracy beliefs (*p* < .001) – experienced greater COVID-19 related media-induced secondary trauma. Educational level, faith in media, and critical thinking did not significantly predict media-related trauma.

**Table 3.** Factors associated with media induced trauma during the pandemic.

Step	Predictors	B	SE B	β	95% CI		*p*	Adjusted R^2^
Step 1	Age***	-.28	.03	-.22	-.35	-.20	<.001	.10
	Gender***	-3.63	.93	-.12	-5.46	-1.80	<.001	
	Education	-.52	.45	-.00	-1.42	.37	.255	
Step 2	Perceived seriousness***	3.12	.49	.23	2.15	4.10	<.001	.14
	Faith in media	-.65	.42	-.01	-1.48	.17	.840	
Step 3	Critical thinking	.06	.06	.03	-.05	.18	.268	
	Belief in conspiracies***	.22	.03	.22	.16	.29	<.001	.19
DV	Media induced trauma							

*Note:* *** = *p* < .001.

To test the robustness of these results we reproduced the overall model using two alternative bootstrapping techniques. Our first bootstrapped analysis resampled 1000 iterations of the full sample with replacement. This reproduced the same results as our initial regression model (see Table S1 in the Supplementary Analyses). Second, it is possible that our large sample resulted in the identification of false positive results. To mitigate this, we conducted an additional bootstrapped analysis where we placed constraints on the sample size of our re-sampled sub-samples. We conducted a power analysis (using G*Power) to determine the size of these sub-samples. Our power analysis revealed that a sample of *n* = 725 was required in order to detect a small effect *f* ^2^ = 0.02 with 80% power. Thus in our second bootstrapped analysis we set *n* = 725 as the sample size for our re-sampled sub-samples. This second bootstrapped analyses largely reproduced the results of the main analysis with one notable difference, critical thinking emerged as a significant predictor in this model (see Table S2 in the Supplementary Analyses).

Second hierarchical regression was administered to assess factors (sample characteristics, perceived seriousness of the pandemic, faith in media, and media-induced trauma) which influence participants’ attitude towards compliance with outbreak related safety guidelines. Collinearity statistics were checked using Tolerance (range = 0.80 – 0.94), and VIF (range = 1.06 – 1.19) values. The models significantly explained 22% (Adjusted *R*^2^ = 0.216) of the variance in compliance related behavior. Demographic characteristics significantly explained only 1% of the variance *F*(3, 911) = 4.19, *p* = .006. The second model with perceived seriousness of the pandemic and faith in media, significantly explained a large additional variance of 19% in compliance behavior *F*(5, 909) = 46.40, *p* < .001. Media-induced trauma, entered at the third step, explained only 2% variance *F*(6, 908) = 43.05, *p* < .001. Individual coefficients in the final model suggested that higher level of education (*p* = .03), higher perceived seriousness of the pandemic (*p* < .001), greater faith in media during the pandemic (*p* = .001), and higher levels of media-induced secondary trauma (*p* < .001) were significantly associated with compliance to safety regulations (see Table 4).

**Table 4.** Factors associated with compliance during the pandemic.

Step	Predictors	B	SE B	β	95% CI		*p*	Adjusted R^2^
Step 1	Age	-.002	.003	-.01	-.008	.004	.457	.10
	Gender	-.08	.07	-.01	-.21	.05	.239	
	Education**	-.09	.03	-.06	-.16	-.02	.005	
Step 2	Perceived seriousness***	.41	.03	.35	.34	.48	<.001	.19
	Faith in media**	.08	.02	.11	.03	.14	.002	
Step 3	Media induced trauma***	.01	.002	.14	.006	.01	<.001	.21
DV	Compliance							

*Note:* ** *p* < .01, *** *p* < .001.

As with the previous analysis, we conducted two follow-up bootstrapped analyses to test the robustness of our results. The first bootstrapped analysis (resampling 1000 iterations of the full sample with replacement), produced the same results as the initial regression (see Table S3 in the Supplementary Analyses). Again, we conducted a second bootstrapped analysis with a smaller sub-sample. Power analyses revealed that a sample of *n* = 688 was required to detect a small effect *f* ^2^ = 0.02 with 80% power. Our second bootstrapped analysis (with re-sampled sub-samples of *n* = 688) produced similar results to the initial analysis with one notable difference, education level was no longer a significant predictor of compliance (see Table S4 in the Supplementary Analyses).

The final hierarchical regression examined whether sample characteristics, (entered at step 1) perceived seriousness of the pandemic, faith in media (entered at step 2), and media-induced trauma (entered at step 3) significantly predicted levels of paranoia during the pandemic. Collinearity statistics were checked using Tolerance (range = 0.81 – 0.94), and VIF (1.05 – 1.23) values. The models were significant explaining a total of 19% (Adjusted *R*^2^ = 0.19) variance. Demographic variables accounted for approximately 8% (Adjusted *R*^2^ = 0.076) of the variance in paranoia levels *F*(3, 828) = 23.86, *p* < .001. Perceived seriousness of the pandemic and faith in media accounted for a very small additional variance of 0.2% *F*(5, 826) = 14.76, *p* < .001. Media-induced trauma significantly explained about 11% of the additional variance in paranoia levels *F*(6, 825) = 33.41, *p* < .001 (see Table 5). Individual coefficients of the final model showed that people who were younger (*p* < .001), were males (*p* < .001), were less educated (*p* = .01), scored lower on perceived seriousness of the pandemic (*p* = .007), and had higher COVID-19 related media-induced secondary trauma, (*p* < .001) had greater levels of paranoia during the pandemic.

**Table 5.** Factors associated with paranoia experiences during the pandemic.

Step	Predictors	B	SE B	β	95% CI		*p*	Adjusted R^2^
Step 1	Age***	-.33	.04	-.15	-.42	-.23	<.001	.07
	Gender*	2.63	1.13	.12	.41	.48	.020	
	Education*	-1.41	.56	-.08	-.2.52	-.30	.012	
Step 2	Perceived seriousness*	-.25	.61	-.01	-1.47	.95	.67	.07
	Faith in media	-.60	.52	-.04	-1.62	.42	.249	
Step 3	Media induced trauma***	.43	.04	.36	.35	.51	<.001	.19
DV	Paranoia							

*Note:* * *p* < .05, *** *p* < .001.

As with compliance and media induced trauma, we conducted two follow-up bootstrapped analyses to test the robustness of the results. The first bootstrapped analysis (resampling 1000 iterations of the full sample with replacement), produced the same results as the initial regression (see Table S5 in the Supplementary Analyses). Power analyses revealed that a sample sample of *n* = 688 was required to detect a small effect *f* ^2^ = 0.02 with 80% power, thus our second bootstrapped analysis re-sampled sub-samples of *n* = 688. Again this produced similar results to the initial analysis with one notable difference, education level was no longer a significant predictor of compliance (see Table S6 in the Supplementary Analyses).

## Discussion

We found that greater frequency of consumption of pandemic related news and believing in conspiracy theories increased likelihood of media-induced secondary trauma. In terms of behavioral and psychological implications, media induced secondary trauma was associated with both compliance towards safety measures and paranoid ideation in the public. However, it is important to note that it was a much stronger predictor of paranoia.

The present study explored psychological health implications of news media consumption during COVID-19 pandemic. Witnessing negative events through news can cause media-induced secondary trauma, which bears resemblance to the traditional diagnosis of Post-traumatic Stress Disorder (American Psychological Association, 2013, Pinchevski, 2016). The findings offering an exploratory international perspective, suggest that in the summer of 2020, most (61%) people consumed news and information about the pandemic either once a day or several times a day. The findings are also in line with recent global statistics suggesting increased news consumption during the pandemic (Watson, 2020a; 2020b). This is concerning as greater news exposure has been previously linked to higher anxiety and lower optimism (McNaughton-Cassill, 2001).

In terms of choice of platforms, data suggests that most people preferred to read about COVID-19 either through online news websites/applications (61%) or social media (61%). Fewer people accessed news via traditional media sources such as television or radio. This finding also supports recent statistics suggesting that more than 70% of the adults accessed news from their smartphones during the pandemic (Watson, 2020c), pointing towards greater new media consumption, i.e. via news applications and social media. It is important to note however that most participants in the present study were young adults, who are more likely to use new forms of news media. Despite recommendations on following more reliable sources (McGuire, 2020), growing concerns related to fake news and misinformation (Pennycook et al., 2020), and constant reports on COVID-19 related myths versus facts (World Health Organization, 2020a, World Health Organization, 2020b) – the present study suggests that globally fewer people accessed WHO (17%) or local governmental website/application (17%) for news about the pandemic.

### Factors related to media induced secondary trauma

The findings suggest that *greater consumption of COVID-related news* increases likelihood of developing media-induced secondary trauma. Isolation due to social distancing and stay-at-home orders, could have also led to higher consumption of media (Freeman et al., 2020) and a forthcoming ‘apocalyptic’ feeling (Inayatullah, 2020). In fact, a study administered in Iran showed that the risk of developing a psychological problem was 1.5 times higher during quarantine (Zakeri et al., 2021). Participants who accessed COVID-19 news directly via *social media* were most likely to experience pandemic related media-induced secondary trauma. This finding supports recent studies suggesting that greater use of social media and newer forms of media, in comparison to traditional media, contributed to negative mental health during the pandemic (Chao et al., 2020, Gao et al., 2020). However, given social media is used heavily to disseminate health information as well (Chandrasekaran et al., 2020), it is important to create conscious consumers who would regulate their news consumptions patterns.

We further found that the use of *WHO* as a source of news for COVID-19 increased likelihood of risk of media-induced trauma. Generally credible sources like WHO are recommended, linked to responsible reporting, and are not expected to contribute towards poorer psychological health (McGuire, 2020). Perhaps anxiety provoking COVID-19 statistics and speculations of declining faith in the institution (Buranyi, 2020), contributed towards greater WHO related psychological distress. However, these findings should be interpreted with caution as these are based on weak correlations (r = 0.02) and few (16.3%) participants accessed news via WHO, majority of whom were younger people in the sample. Also, given younger people accessed WHO more and are also more likely to score higher on media trauma and paranoia as shown in the current study, age might be a confounding variable in explaining WHO news being related to media-trauma. With respect to age, it is important to note that since younger people were more likely to experience worse moods (Ingram et al., 2022) and greater stress (Horesh et al., 2020), such factors could also be driving higher scores in secondary media trauma.

In contrast, participants who consumed news via *radio* displayed lower likelihood of developing media-induced secondary trauma. Lack of disturbing visuals and emphasis on local rather than global news on the radio may have contributed towards lower secondary trauma.

In terms of factors predicting COVID-19 media induced secondary trauma, it was found that *females* and *younger people* were at a greater risk, both are groups that have previously been known to have higher vulnerability to PTSD (Norris et al., 2002). This finding is supported by a research administered in Israel which found that males and older people experienced less mental health problems during the pandemic (Horesh et al., 2020). Demographic factors explained the maximum variance in the model, with age being a stronger factor. Given younger people are more likely to be ‘Cyberchondriacs’, they may repeatedly check symptoms online as a safety-seeking behavior (Jungmann and Witthöft, 2020), and this may have put them at a risk of developing greater media-induced trauma. Results also confirmed that people who perceived the *pandemic to be more serious* were also more likely to experience secondary trauma while consuming news.

While believing in conspiracies has been previously linked to higher distress and anxiety (Chen et al., 2020), the present study shows that people who tend to generally believe more in conspiracies are also at a greater risk of experiencing pandemic related media trauma. To our knowledge this is the first study that has examined this association. Interestingly higher educational qualifications, increased faith in media, greater ability to think critically or ability to identify credible sources of news did not appear to be protective against media related secondary trauma.

### The role of secondary trauma in influencing both compliance and paranoia

Media also plays an important role in spreading awareness regarding safety measures. Compliance to safety regulations has been a challenge during the pandemic. Findings suggest that people with higher *educational qualifications* are more likely to comply with safety regulations. Results further reiterate that *perceiving the pandemic to be serious* and credible and having greater *faith in media* contribute to greater compliance to safety regulations. In line with recent research, the findings suggest that along with depicting the ‘seriousness’ of the pandemic, campaigns promoting safety regulations, should focus on building and maintaining public’s trust in the news and information being presented to them (Koning et al., 2021). Interestingly *media-induced trauma* also increased likelihood of compliance to safety regulations, implying that emotionally distressing content in the news contributes to the public’s compliance. However, this finding should be interpreted with caution as it explained a very small variance.

Finally, we examined the strength of association between news related secondary trauma and public’s levels of paranoia during the pandemic. It was found that *younger population*, *males*, and people with lower levels of *education* displayed higher levels of paranoia during the pandemic. While certain personality attributes may be related irrespective of the health emergency, future studies should explore these findings in the context of the pandemic. Age related finding is especially concerning as recent research has shown that being suspicious of others, indicating higher levels of paranoia, reduces likelihood of compliance to safety measures (Koning et al., 2021). *Perceiving the pandemic to be serious* was found to be significantly associated with lower levels of paranoia in the public, when media-induced trauma was entered into the analysis, however it explained a small variance and effect size. Importantly, experiencing COVID-19 related *media trauma* was a strong predictor of higher levels of paranoia in the public. Generally, feelings of paranoia include grandiose delusions, feelings of lack of control, feelings of suspicion, cynicism, or hostility towards others. It becomes part of how we perceive social relations around us, and can be highly distressing (Fenigstein and Vanable, 1992). Moreover, such feelings tend to reduce institutional trust and likelihood of adherence to safety campaigns (Koning et al., 2021). It is possible that exposure to coronavirus-related negative imagery via media such as graphics showing death rates, overcrowded cemeteries, overwhelmed healthcare workers, and hearing conspiracy theories regarding origin and spread of the virus may have contributed towards feelings of higher paranoia in the public during the pandemic.

### Implications, limitations, and future directions

Largely, this finding supports recent but a growing body of literature suggesting that the pandemic has had severe mental health implications (Odriozola-González et al., 2020; Usher et al., 2020; World Health Organization, 2020a; Zysberg and Zisberg, 2020). In fact, the implications could be more severe for individuals with pre-existing mental health conditions (Mensi et al., 2021) or vulnerable populations (Fiskin, 2021). With countries going under second or third phases of lockdown, the mental health implications of pandemic related news need to be closely understood. While previous research has established the role played by personal trauma in making people paranoid (Gracie et al., 2007), our study discusses the pervasive nature of secondary trauma in creating a similar effect.

It is also important to note that while media-induced secondary trauma increases likelihood of both compliance to safety measures and levels of paranoia, results imply that it has a much greater impact on levels of paranoia in the public. Similar to recent previous research, findings suggest that while fear may facilitate compliance to safety measures, heightened fear or anxiety may have a significant negative impact on mental health (Lin et al., 2020). It is also important to note that the use of fear as an extrinsic motivator of behavior can be questionable with regards to its long-term effectiveness (Marks, 1973), as continued exposure to pandemic-related media may eventually lead to the development of resistance and further apprehension towards public health guidelines. Fear-based strategies used by media may have been somewhat effective in creating an initial avoidance-based response during the pandemic (Mobbs et al., 2015), however findings further suggest that these may have worked at the cost of public’s psychological health. It is media’s responsibility to be ethical and sensitive in presentation of the news. It is also health safety official’s responsibility to balance out fear messaging for safety measures in order to minimize psychological risks.

The study further offers an expanded understanding of the theory of *vicarious traumatization*, as it provides further evidence for its negative impact in the form of increased paranoia and potentially mistrust in the general public. This awareness is of particular relevance, given that public health messaging sometimes while being effective, can contribute to secondary traumatic stress. The present study has shown that not only our media consumption, especially during such health emergencies, can increase our secondary trauma, it may further change how we experience self, others, and the world around us (Sui and Padmanabhanunn, 2016). Our findings inform psychological support services, acknowledging the impact of secondary trauma during the pandemic.

Given the nature of the pandemic, it is important to explore behavioral and psychological aspects at an international level, in addition to having a localized community perspective. The current study explores emotional responses which are potentially universal in nature and could be used to facilitate interventions in diverse settings. It is also important to note that the current study was administered when most countries were under a lockdown, indicating a more severe behavioral and psychological impact (Zakeri et al., 2021).

The sample size and a wide representation of participants from countries which are usually underrepresented in international research are important strengths of the study. Research scholars have discussed about the need for greater representation of COVID-19 related research from understudied regions such as the Global South (Malherbe and Niekerk, 2020). To our knowledge, this is the first study to have explored COVID-19 related media-induced secondary trauma and its behavioral and psychological implications. However, measurement of media related trauma is not a clinical assessment and should be interpreted with caution. The self-reported questionnaire provides information on participant’s perceived secondary media trauma. Findings also need to be generalized with caution due to use of convenience and snowball sampling, an unequal global representation, and use of one-item Likert scale questions for a few variables. Future research could examine media-induced trauma in national samples and in news reporters, especially during health crises.

The effect sizes reported in the current research are relatively small, potentially limiting the applicability of this work (R^2^ for full models = 0.19, 0.21, and 0.19). We note however that these effect sizes are comparable to existing research in the wider COVID-19 related literature (e.g., McHugh et al., 2023, Van Bavel et al., 2022). Despite these small effects the current research provides important insights into the possible links between media consumption and negative outcomes such as belief in conspiracy theories, and media induced trauma. It is important to note that our correlational design means we cannot determine causality or the direction of these effects. Future research using alternative methods should examine these findings in more detail to better inform our understanding of the processes involved.

## Conclusion

The international community may have been overwhelmed by the sheer amount of information during the coronavirus outbreak and warned of a subsequent ‘infodemic’, further leading to negative mental health implications. The present study highlights the role of various psycho-social dimensions which act as risk factors for developing vicarious trauma from news media. It also promotes collective wellbeing beyond specific geographical locations.

Important findings show that frequency of media consumption and use of social media for news increased the risk of developing media-generated secondary trauma. Moreover, people who tend to generally believe in conspiracies also suffered from greater media-induced secondary trauma during the pandemic. Unhealthy media usage during the pandemic increase likelihood of paranoid ideation, cynicism, and mistrust. In terms of compliance, believing the pandemic to be credible and having trust in news media facilitated people to follow safety guidelines more than others in their community. While media-induced trauma somewhat positively contributed towards compliance to safety regulations, it also significantly increased levels of paranoia in the public. Therefore, future research should explore media’s responsibility towards emotionally distressing news and fear-based health messaging. It is important to create awareness related to psychological costs of media delivery and consumption and curate healthy patterns of engagement with news or social media feeds.

## Supplemental Material

Supplemental Material - Mental health hygiene during a health crisis: Exploring factors associated with media-induced secondary trauma in relation to the COVID-19 pandemic Click here for additional data file. Supplemental Material for Mental health hygiene during a health crisis: Exploring factors associated with media-induced secondary trauma in relation to the COVID-19 pandemic by Nishtha Lamba, Olga Khokhlova, Aditi Bhatia and Cillian McHugh in Health Psychology Open

## Data Availability

The data given this article are data is available at https://osf.io/a5hpy/?view_only=c11401295a8846fbb3af9d52cbc2539f
