# Supplementary material for: Mental health hygiene during a health crisis: Exploring factors associated with media-induced secondary trauma in relation to the COVID-19 pandemic
Source: Health Psychol Open. 2023 Sep 22;10(2):20551029231199578. doi: 10.1177/20551029231199578 (PMC10517610; doi:10.1177/20551029231199578)
Supplement: Supplemental Material - Mental health hygiene during a health crisis: Exploring factors associated with media-induced secondary trauma in relation to the COVID-19 pandemic [file sj-pdf-1-hpo-10.1177_20551029231199578.pdf]

Age

For Peer Review

Supplementary Analyses

Table S1

Bootstrapped predictors of media induced trauma

	**Estimate**	**95% CI**		***p***
(Intercept)	23.48	15.16	31.87	< .001**
Age	-0.24	-0.32	-0.18	.001*

https://mc.manuscriptcentral.com/hpo

Gender	-3.43	-5.15	-1.55	.001*
Education	-0.07	-0.91	0.77	.891
Perceived seriousness	3.36	2.34	4.33	< .001**
Faith in media	-0.20	-1.01	0.60	.616
Critical thinking	0.07	-0.06	0.19	.279
Belief in conspiracies	0.23	0.17	0.29	< .001**

For Peer Review

Table S2

Reduced sub-sample (n = 725) bootstrapped predictors of media induced trauma

	**Estimate**	**95% CI**		***p***
(Intercept)	27.30	13.11	32.33	< .001**
Age	-0.22	-0.32	-0.17	< .001**
Gender	-4.87	-5.23	-1.71	< .001**
Education	-0.43	-0.91	0.92	.346

Perceived seriousness	3.04	2.27	4.37	< .001**
Faith in media	-0.46	-1.17	0.79	.299
Critical thinking	0.13	-0.07	0.21	.046*
Belief in conspiracies	0.19	0.15	0.30	< .001**

For Peer Review

Table S3

Bootstrapped predictors of compliance

	**Estimate**	**95% CI**		***p***
(Intercept)	1.75	1.28	2.21	< .001**
Age	0.00	0.00	0.01	.784
Gender	-0.03	-0.16	0.10	.693
Education	-0.06	-0.12	0.00	.046*

Perceived seriousness	0.38	0.31	0.45	< .001**
Faith in media	0.10	0.04	0.16	< .001**
Media induced trauma	0.01	0.01	0.02	< .001**

For Peer Review

Table S4

Reduced sub-sample (n = 725) bootstrapped predictors of compliance

	**Estimate**	**95% CI**		***p***
(Intercept)	1.33	1.14	2.26	< .001**
Age	0.00	-0.01	0.01	.603
Gender	-0.02	-0.15	0.13	.819
Education	-0.03	-0.12	0.01	.380

Perceived seriousness	0.47	0.29	0.47	< .001**
Faith in media	0.07	0.03	0.16	.027*
Media induced trauma	0.01	0.01	0.02	< .001**

For Peer Review

Table S5

Bootstrapped predictors of paranoia

	Estimate	95% CI		*p*
(Intercept)	42.52	34.68	50.76	< .001**
Age	-0.23	-0.33	-0.13	.001*
Gender	4.81	2.79	6.95	< .001**
Education	-1.09	-2.17	-0.06	.043*
Perceived seriousness	-1.54	-2.77	-0.36	.016*

Faith in media	-0.38	-1.37	0.65	.417
Media induced trauma	0.44	0.36	0.53	< .001**

For Peer Review

Table S6

Reduced sub-sample (n = 725) bootstrapped predictors of paranoia

	Estimate	95% CI		*p*
(Intercept)	43.68	34.47	51.14	< .001**
Age	-0.26	-0.32	-0.14	< .001**
Gender	5.17	2.30	7.13	< .001**
Education	-0.66	-2.07	-0.16	.229

Perceived seriousness	-2.39	-2.80	-0.21	< .001**
Faith in media	-0.50	-1.50	0.79	.317
Media induced trauma	0.48	0.35	0.53	< .001**

For Peer Review
